# Clinical characteristics and outcomes of patients with severe acute respiratory infections (SARI): results from the Egyptian surveillance study 2010–2014

**DOI:** 10.1186/s40248-019-0174-7

**Published:** 2019-04-01

**Authors:** Ashraf Hatem, Sherif Mohamed, Usama E. Abu Elhassan, Eman A. M. Ismael, Magda S. Rizk, Amany El-kholy, Mohamed El-Harras

**Affiliations:** 1 Department of Chest Diseases, Faculty of Medicine, Cairo University, Cairo, Egypt; 2 Department of Chest Diseases and Tuberculosis, Faculty of Medicine, Assiut University, Assiut, 71516 Egypt; 3 Department of Anesthesia and Intensive Care, Faculty of Medicine, Cairo University, Cairo, Egypt; 4 Department of Clinical Pathology, Faculty of Medicine, Cairo University, Cairo, Egypt; 5 Department of Clinical Pathology, Faculty of Medicine, Mansura University, Mansura, Egypt

**Keywords:** Clinical, Outcomes, Viral, SARI, Egypt, Surveillance

## Abstract

**Background:**

Respiratory viral and atypical bacterial infections data in Egyptian patients are sparse. This study describes the clinical features and outcomes of patients with severe acute respiratory infections (SARI) in hospitalized patients in Egypt.

**Methods:**

SARI surveillance was implemented at Cairo University Hospital (CUH) during the period 2010–2014. All hospitalized patients meeting the WHO case definition for SARI were enrolled. Nasopharyngeal/oropharyngeal (NP/OP) swabs were collected and samples were tested using RT-PCR for influenza A, B, respiratory syncytial virus (RSV), human metapneumovirus (hMPV), parainfluenza virus (PIV 1,2,3,4), adenovirus, bocavirus, coronavirus, enterovirus, rhinovirus, and atypical bacteria. Data were analyzed to calculate positivity rates for viral pathogens and determine which pathogens related to severe outcomes or resulted in death.

**Results:**

Overall, 1,075/3,207 (33.5%) cases had a viral etiology, with a mean age of 5.74 (±13.87) years. The highest rates were reported for RSV (485 cases, 45.2%), PIV (125, 11.6%), and adenovirus (105, 9.8%). Children had a higher viral rate (981, 91.2%) compared to 94 (8.8%) cases in adults. Patients with identified viruses had significantly lower rates for ICU admission, hospital stay, mechanical ventilation, and overall mortality than those without identified viruses. No infections were independently associated with severe outcomes.

**Conclusions:**

Viral pathogens were encountered in one-third of hospitalized adult and pediatric Egyptian patients with SARI, while atypical bacteria had a minor role. Highest rates of viral infections were reported for RSV, PIV, and adenovirus. Viral infections had neither negative impacts on clinical features nor outcomes of patients with SARI in our locality.

## Background

The World Health Organization (WHO) estimates that acute respiratory infections (ARI) cause annual deaths approaching 4 million, at a rate of more than 60 deaths/100,000 populations [1]. Viruses are responsible for 30-70 % of ARI where respiratory syncytial virus (RSV), influenza virus, parainfluenza virus (PIV), human Bocavirus, human metapneumovirus (hMPV), adenovirus, rhinovirus, enterovirus and Coronaviruses account for the majority of these cases [1, 2]. The 2009 influenza pandemic had highlighted the need for more global data on severe influenza disease, so the WHO recommended conducting surveillance for hospitalized severe acute respiratory infection (SARI), as well as influenza-like illness (ILI) in outpatients [3–6]. SARI surveillances are now conducted in many countries around the world; however, because of limited resources, they are only conducted in limited settings in the Middle East and Egypt [7–9]. Furthermore, the role of individual viral or atypical bacterial infection in causing ARI is not usually documented [10, 11].

In the current study, we analyzed surveillance data from Egyptian patients with SARI, enrolled at Cairo University Hospital (CUH) from 2010 to 2014. We aimed to calculate proportions of positive samples for different viral pathogens, to determine which pathogens were related to severe outcomes, and to address the impact of SARI on the clinical outcomes of enrolled patients, in terms of morbidity and mortality.

## Methods

### Study population

Cairo University Hospital (CUH) is a 5100-beds tertiary referral teaching hospital. Inclusion criteria consisted of hospitalized adults (defined as age ≥ 18 years old), as well as pediatric patients (age < 18 years old), with the diagnosis of SARI, who provided a respiratory sample, from February 2010 to February 2014. Due to an annual review by dedicated investigators and updates to WHO guidelines, the case definition for SARI has evolved over the study period. Before February 2010, as a global-surveillance case definition of SARI did not exist, the definition for SARI was adapted from the WHO protocol on rapid response for persons ≥5 years old [3]. Whereas, for children <5 years old, SARI definition was adapted from the program for Integrated Management of Childhood Illness [4]. After March 2011, the global standards and tools for influenza surveillance developed by the WHO were adopted [5]. As of January 2014, the WHO surveillance case definitions for SARI was implemented [6] as follows, acute respiratory infection with history of fever or measured fever of ≥ 38 C°; and cough; with onset within the last 10 days; and requiring hospitalization [6]. An enrollment form was used to collect data from enrolled eligible patients including patient demographics, medical history, clinical signs and symptoms, comorbidities, reported influenza vaccine status, recent travel history, treatment, clinical course, and outcome. Patients with incomplete medical records were excluded.

### Clinical samples and viral detection techniques

Nasopharyngeal (NP) and oropharyngeal (OP) swabs for detecting viruses and blood cultures for detecting bacteria were taken from eligible patients on admission using operating procedures described by the WHO [12]. Specimens were taken an average of 7 days after illness onset (range: 1–66 days).

Total nucleic acid (TNA) was extracted by the automated KingFisher Flex Magnetic Particle Processor (Thermo Scientific, Waltham, MA, USA) using MagMAX Total Nucleic Acid Isolation Kit (Cat No. AM 1840, Applied Biosystems, Foster, CA, USA) according to the manufacturer’s instructions. The viral target was amplified using specific primers and probes produced by the CDC (Atlanta, GA, USA) and following standard protocol for reverse transcription polymerase chain reaction detection. From 2010 to 2012, testing for RSV, adenovirus, human parainfluenza viruses (hPIV) 1, 2 and 3, influenza (A and B) and human metapneumovirus was conducted at CUH laboratory and sent for confirmation by the Naval Medical Research Unit No.3 (NAMRU-3) laboratory. From 2013 to 2014, testing was conducted at CUH laboratory. For all samples, the human RNase P gene (RP) was tested as an internal positive control to ensure proper sample collection and nucleic acid extraction. Samples were considered positive to the viral target if the amplification curve crossed the threshold line before cycle 40. All clinical samples should be positive to RP with cutoff value ≤ 37, as prescribed previously [8]. Blood samples were collected for detection of Mycoplasma pneumonia, Chlamydia pneumonia, and Legionella pneumophila, using RT-qPCR.

### Ethical standards

Prior to study initiation, the study protocol was reviewed and approved by Institutional Review Board at the NAMRU-3, as well as the ethical committee of CUH, in compliance with all applicable federal U.S. regulations governing the protection of human subjects. An informed written consent was obtained from the patients (in the case of adult patients) or patients’ parent/legal guardian (in the case of pediatric patients).

### Statistical analysis

Data analyses were conducted using the software SPSS (Statistical Package for the Social Science; IBM Corp, NY, USA); version 22. Data were summarized using median (range) for quantitative variables and number and percent for qualitative variables. Comparison between groups was done using the Chi-square test for qualitative variables, independent sample t-test for normally distributed quantitative variables, while the Mann-Whitney U test was used for quantitative variables that are not normally distributed. Indicators of severe disease were assessed for each pathogen of interest using Mantel-Haenszel estimates to calculate odds ratios and confidence intervals and the Mantel-Haenszel chi-squared test to assess statistical significance [13]. Logistic regression was used to examine associations between viral respiratory pathogens and severe outcomes, defined as illness requiring ventilation or intensive care unit (ICU) or resulting in death while controlling for demographic and clinical characteristics. Only variables with statistically significant univariate association with severe outcomes were included in multivariate regression analysis. All tests were two-sided, and differences with *p* <0.05 were considered significant*.*

## Results

### Demographic data of the study population

Out of 3,207 participants enrolled in this SARI surveillance, 1,075 (33.5%) had positive results for viral and atypical bacteria tested. They included 569/1,075 (53%) females and 506/1,075 (47%) males. The median age was one year (range 0-85 years). Children less than 18 years had a higher viral etiology (981 patients, 91.2%) compared to 94 (8.8%) ones in adults. Notably, children <5 years represented 83% of patients. The highest rates of viral infections were reported for RSV (485 patients, 45.2%), PIV (125, 11.6%), and adenovirus (105, 9.8%). Other encountered viruses included rhinovirus, hMPV, and BOCA virus (2%,7%, and 1%, respectively). Only 3 cases were positive for Mycoplasma and were co-infected with RSV, while only one case of Chlamydia was co-infected with RSV and hMPV. Neither Coronavirus nor Legionella was detected. Table 1 shows these data.

**Table 1:** Demographic and clinical characteristics of patients hospitalized with severe acute respiratory infection (SARI) cases in Egypt, 2010–2014

Characteristic	Viruses Not Identified (n = 2,132) N (%)	Enrolled SARI cases (n = 1,075) N (%)	P*	RSV (n = 485) N (%)	P^$^	Multiple Viruses (n = 174) N (%)	P^$^	PIV (n = 125) N (%)	P^$^	Adeno-Virus (n = 105) N (%)	P^$^	Influenza Viruses (n = 77) N (%)	P^$^
Demographics													
Gender													
Female	810 (38)	569 (53)	0.562	257 (53)	1.000	90 (52)	0.741	65 (52)	0.449	47 (45)	0.081	48 (62)	0.054
Male	1,322 (62)	506 (47)		228 (47)		84 (48)		60 (48)		58 (55)		29 (38)	
Age in years													
Mean ± SD	16.96 ± 25.3	5.74 ± 13.8		3.15 ± 9.64		5.48 ± 12.55		6.48 ± 14.82		4.48 ± 11.12		19.52 ± 23.47	
Median	1.0	1.0		0.67		1.0		1.0		1.0		5.0	
(Range)	(0–90)	(0–85)		(0–85)		(0–77)		(0–74)		(0–57)		(0–76)	
< 18 years	1,493 (70)	981 (91)	0.000	470 (97)	0.000	162 (93)	0.143	111 (89)	0.523	97 (92)	0.330	47 (61)	0.000
< 1 year	831	501		269		73		59		29		23	
1–5 years	567	384		165		66		46		52		17	
> 5 years	95	96		36		23		6		16		7	
> 18 years	639 (30)	94 (9)		15 (3)		12 (7)		14 (11)		8 (8)		30 (39)	
Signs & symptoms at presentation													
Shortness of breath	1,555 (73)	1,033 (96)	0.000	485 (100)	NA	174 (100)	NA	125 (100)	NA	105 (100)	NA	77 (100)	NA
Sore throat	654 (31)	273 (25)	0.001	174 (36)	0.001	35 (20)	0.087	33 (26)	0.429	18 (17)	0.024	18 (23)	0.394
Sputum production	1,169 (55)	566 (53)	0.077	254 (52)	0.902	86 (49)	0.396	66 (53)	0.524	63 (60)	0.068	46 (60)	0.120
Hemoptysis	62 (3)	20 (2)	0.047	5 (1)	0.110	4 (2)	0.869	3 (2)	0.426	2 (2)	0.599	3 (4)	0.175
Body aches	153 (7)	165 (15)	0.000	60 (12)	0.005	30 (17)	0.490	26 (21)	0.060	22 (21)	0.065	10 (13)	0.391
Tachypnea	1,732 (81)	1,000 (93)	0.000	442 (91)	0.362	154 (88)	0.292	108 (86)	0.459	98 (93)	0.084	74 (96)	0.038
Nasal congestion	300 (14)	647 (60)	0.000	322 (66)	0.161	113 (65)	0.244	69 (55)	0.128	53 (50)	0.397	30 (39)	0.131
Wheezing	360 (17)	881 (82)	0.000	414 (85)	0.186	141 (81)	0.342	108 (86)	0.123	82 (78)	0.455	42 (55)	0.000
Stridor	22 (1)	7 (0.6)	0.127	3 (0.6)	1.000	0 (0)	0.606	1 (0)	0.586	1 (0)	0.514	2 (0)	0.052
Abnormal Breath Sounds	955 (45)	558 (52)	0.159	239 (49)	0.140	84 (48)	0.314	74 (59)	0.054	60 (57)	0..256	49 (64)	0.023
Nausea or vomiting	209 (10)	116 (11)	0.360	54 (11)	1.000	29 (17)	0.011	12 (10)	0.349	8 (7)	0.174	5 (6)	0.401
Convulsions	63 (3)	108 (10)	0.000	44 (9)	0.245	18 (10)	0.891	15 (12)	0.284	17 (16)	0.026	8 (10)	0.215
Comorbidities													
Chronic Resp disease	343 (16)	460 (43)	0.002	227 (47)	0.062	52 (30)	0.044	35 (28)	0.066	45 (43)	0.078	35 (45)	0.053
Asthma	87	119		77		22		21		28		26	
COPD	42	66		13		6		2		0		4	
Bronchiectasis	101	133		88		11		9		12		2	
Others^a^	113	142		49		13		3		5		3	
Cardiac disease	512 (24)	214 (20)	0.392	81 (17)	0.046	37 (21)	0.050	31 (25)	0.078	26 (25)	0.055	12 (16)	0.158
Heart failure	299	44		22		13		14		9		7	
Congenital HD	114	147		48		15		11		15		2	
Cardiomyopathy	99	23		11		9		6		2		3	
Endocrine disease	147 (7)	124 (12)	0.000	50 (10)	0.249	19 (11)	0.897	24 (19)	0.004	13 (12)	0.420	12 (16)	0.158
Diabetes mellitus	100	106		37		12		21		11		11	
Obesity	47	18		13		7		3		2		1	
Neuromuscular disease	176 (8)	90 (8)	0.001	30 (6)	0.033	15 (9)	0.063	19 (15)	0.012	10 (10)	0.398	6 (8)	0.233
Muscle dis	123	37		13		11		12		6		4	
Epilepsy	53	53		17		4		7		4		2	
Renal disease	43 (2)	11 (1)	0.951	5 (1)	0.866	0 (0)	0.322	3 (2)	0.198	0 (0)	0.314	2 (3)	0.200
Chronic RF	34	9		5				3				2	
Nephrotic Syndrome	9	2		0				0				0	
Hepatic disease	54 (3)	9 (1)	0.002	2 (0.5)	0.001	3 (2)	0.192	2 (2)	0.277	1 (1)	0.579	1 (1)	0.496
Ch hepatitis	9	5		2		2		0		1		1	
Liver cirrhosis	33	3		0		1		2		0		0	
Hepatic failure	12	1		0		0		0		0		0	
Hematologic disease	23 (1)	5 (0.5)	0.583	0 (0)	0.051	1 (0.5)	0.428	1 (1)	0.377	2 (2)	0.079	1 (1)	0.496
Clinical course													
Pneumonia	175 (8)	29 (3)	0.004	13 (2.6)	0.033	4 (2)	0.066	1 (1)	0.288	4 (4)	0.054	3 (4)	0.299
Admission to ICU	606 (28)	219 (20)	0.000	82 (17)	0.022	41 (23)	0.123	20 (16)	0.007	22 (21)	0.064	36 (47)	0.045
Mechanical ventilation	221 (10)	100 (9)	0.412	43 (9)	0.052	13 (7)	0.778	8 (6)	0.373	7 (7)	0.213	17 (22)	0.001
Complications													
Respiratory failure	45 (2)	22 (2)	0.033	9 (2)	0.552	4 (2)	0.488	2 (2)	0.607	4 (4)	0.101	1 (1)	0.349
ARDS	3 (0.1)	15 (1.5)	0.011	8 (2)	0.063	4 (2)	0.072	0 (0)	0.237	0 (0)	0.064	0 (0)	0.078
Outcomes^$^													
Discharged	1,852 (87)	956 (89)	0.005	433 (89)	0.045	153 (88)	0.051	116 (93)	0.289	100 (95)	0.131	62 (81)	0.006
Transferred	174 (8)	95 (8.8)		40 (8.6)		20 (11.5)		7 (6)		3 (3)		10 (13)	
Died	106 (5)	24 (2.2)		12 (2.4)		4 (2)		1 (1)		2 (2)		4 (5)	

*P for comparison between virus-infected (SARI-positive) and non-infected (SARI-negative) individuals. P^$^ for SARI patients with a positive result for that pathogen compared to a reference group of tested SARI patients with a negative result for that pathogen. *RSV* Respiratory syncytial virus, *PIV* Para-influenza virus, *ICU* Intensive care unit, *ARDS* Acute respiratory distress syndrome, *NA* Not available ^a^ Others; immotile cilia syndrome, interstitial lung disease

### Clinical characteristics of viral-infected versus no virus-detected individuals

In comparison to non-viral infected individuals, viral-infected SARI ones had significantly predominant signs and symptoms at presentation. Particularly, they had significant viral prodromal symptoms, as well as tachypnea, wheezes, and convulsions (p=0.000 each). Among individual viral pathogens, SARI patients with influenza had more significant tachypnea (p= 0.038), wheezes (p=0.000), and abnormal breath sounds (p= 0.023), than those with non-influenza viral infections. Patients whose specimens were collected within 5 days of the onset of symptoms were more likely to have a viral pathogen detected than those whose specimens were collected later (73% versus 36%, p = 0.047).

Fifty-three percent of patients had at least one underlying medical condition. These comorbidities included chronic respiratory disorders (asthma, COPD, bronchiectasis, and immotile cilia syndrome), cardiac disorders (heart failure congenital heart diseases, and cardiomyopathy), neuromuscular disorders (epilepsy, cerebral palsy, and myopathies), hematological disorders (thalassemia), endocrine disorders (diabetes mellitus, hypothyroidism, and morbid obesity), renal disorders (end-stage renal disease), and liver disorders (liver cirrhosis and hepatic failure).

Patients with comorbidities (n = 570, 53%) were significantly older compared to those with no comorbidities (median age: 54 versus 3, *p* <0.001). Additionally, they were significantly more likely to be symptomatic.

In terms of comorbidities, patients with and without viral detection differed significantly in the frequencies of chronic respiratory (p=0.002), endocrine (p=0.000), hepatic (p=0.002), and neuromuscular disorders (p=0.001). Among individual viral pathogens, SARI patients with para-influenza virus had significant endocrine (p= 0.004), and neuromuscular disorders (p=0.012), than those with non-para-influenza viral infections.

For influenza vaccination history; 832/1,075 (77.4%) cases did not receive the vaccine within the 12 months prior to hospital admission, while 243/1,075 (22.6%) were reported as unknown for an influenza vaccination status. Table 1 details these results.

### Clinical course, complications, and outcomes in viral-infected patients

In comparison to non-viral infected individuals, viral-infected SARI ones had significantly lower rates of pneumonia (p=0.004) and admission to the ICU (p=0.000). Patients with influenza virus tended to have significantly different rates of admission to the ICU (p=0.045), and mechanical ventilation (p=0.001), in comparison to those with non-influenza infections. With regards to complications, viral-infected SARI patients had significant differences for developing respiratory failure (p=0.033), and acute respiratory distress syndrome; ARDS (p=0.011), in comparison to those without viral infections.

Overall mortality in SARI-positive patients was 24/1,075 (2.2%) and peaked at 1% in 2014. Overall, only 2(8%) were adults, while 22 (92%) were children. Among children, 18(75%) were aged <5 years. Overall, two-thirds (16/24) had comorbidities. All patients who died were admitted to the ICU and mechanically ventilated. Notably, all patients who died tested positive for a viral pathogen; twelve were positive for RSV, four for influenza virus, two for adenovirus, one for hMPV, one for PIV and four for mixed viral infections, respectively. Among those who died, there was a significant difference between those with (2.2%) and without (5%) viral detection (*p* = 0.005). Among individual viral pathogens, SARI patients with RSV and influenza had significant deaths (p= 0.045 and 0.006), in comparison to those with non-RSV and non-influenza viral infections. No mortality was reported for patients with atypical bacteria (Table 1).

### Severe outcomes in viral-infected patients

No infections were independently associated with increased severity of SARI, as indicated by illness requiring mechanical ventilation and/or ICU and/or resulting in death. There was strong evidence that individuals with RSV and influenza were less likely to experience a severe outcome than those not infected with each of these pathogens (RSV OR 1.433, 95% CI 4.698-6.132. p=0.021, influenza OR 3.937, 95% CI 2.447-6.3340, p=0.000). Individuals with multiple infections were no more likely than those with infection with a single pathogen to experience severe outcomes (OR 0.232, 95% CI 0.155-0.619, p = 0.240).

When analyses were stratified by age, neither significant differences in severe outcomes could be encountered between viral-infected and non-infected individuals (OR 0.983, 95% CI 0.503-1.924, p=0.961 and OR 1.100, 95% CI 0.704-1.718, p=0.675) nor between individual viral infections, among children and adults. Table 2 shows these details (Data for PIV, hMPV, Boca virus, rhino-, and enterovirus are not shown in the table).

**Table 2:** Indicators of the severity of SARI by pathogen and age

	SARI cases			RSV			Adenovirus			Influenza			Multiple Viruses		
	No (%)	OR (95% CI)	P*	No (%)	OR (95% CI)	P^$^	No (%)	OR (95% CI)	P^$^	No (%)	OR (95% CI)	P^$^	No (%)	OR (95% CI)	P^$^
All participants															
Ventilation	100/ 1,075 (9)	1.280 (0.703- 2.329	0.419	43/485 (9)	0.177 (−1.005- 0.185)	0.866	7/105 (7)	1.293 (0.682- 2.452)	0.431	17/77 (22)	3.123 (1.743- 5.598)	0.000	13/174 (7)	0.755 (0.412- 1.386)	0.365
ICU	219/ 1,075 (20)	0.972 (0.706- 1.337)	0.861	82/485 (17)	1.897 (12.591- 13.635)	0.017	22/105 (21)	1.040 (0.634- 1.707)	0.876	36/77 (47)	3.910 (2.431- 6.290)	0.000	41/174 (23)	0.225 (0.162- 0.612)	0.254
Death	24/ 1,075 (2)	0.00 (0.941- 0.966)	0.557	12/485 (2)	0.530 (−1.215- 0.625)	0.637	2/105 (2)	0.837 (0.194- 3.609)	0.811	4/77 (5)	0.986 (−0.114- 2.085)	0.079	4/174 (2)	0.036 (1.050- 1.122)	0.948
Severe Outcome	219/ 1,075 (20)	0.972 (0.706- 1.337)	0.861	82/485 (17)	1.433 (4.698- 6.132)	0.021	22/105 (21)	1.047 (0.638- 1.738)	0.857	36/77 (47)	3.937 (2.447- 6.334)	0.000	41/174 (23)	0.232 (0.155- 0.619)	0.240
Children <18 years															
Ventilation	66/ 981 (7)	1.032 (0.305- 3.541)	0.952	36/485 (8)	0.894 (0.566- 1.413)	0.632	7/105 (7)	0.907 (04.05- 2.032)	0.812	5/77 (6)	1.785 (0.676- 4.713)	.0243	13/174 (7)	0.995 (0.536- 1.849)	0.988
ICU	178/ 981 (18)	0.983 (0.503- 1.924)	0.961	68/485 (14)	0.838 (0.594- 1.182)	0.314	11/105 (10)	0.691 (0359- 1.330)	0.268	10/77 (13)	2.008 (0.952- (4.237)	0.067	32/174 (18)	1.418 (0.921- 2.185)	0.113
Death	13/ 981 (1)	0.973 (0.960- (0.987)	0.784	12/485 (2)	1.363 (0.583- 1.385)	0.474	2/105 (2)	0.966 (0.222- 4.198)	0.963	2/77 (2)	2.640 (0.594- 11.740)	0.202	4/174 (2)	1.162 (0.338- 3.479)	0.789
Severe Outcome	178/ 981 (18)	0.983 (0.503- 1.924)	0.961	68/485 (14)	0.849 (0.602- 1.199)	0.353	11/105 (10)	0.697 (0.362- 1.342)	0279	10/77 (13)	2.024 (0.959- 4.271)	0.064	32/174 (18)	1.432 (0929- 2.206)	0.114
Adults >18 years															
Ventilation	34/94 (36)	1.357 (0.645- 2.856)	0.421	7/485 (1)	0.966 (0.114- 2.085)	0.078	0/105 (0)	2.991 (0.851- 10.514)	0.088	12/77 (16)	2.878 (1014- 8.166)	0.052	0/174 (0)	0.787 (0.669- 0.885)	0.063
ICU	41/94 (44)	1.100 (0.704- 1.718)	0.675	14/485 (3)	1.493 (0.282- 2.452)	0.527	11/105 (10)	3.235 (0.671- 15.593)	0.143	26/77 (34)	0.929 (0.392- 2.198)	0.866	9/174 (5)	0.606 (0.203- 1.815)	0.371
Death	11/94 (12)	0.934 (0.912- 0.965)	0.793	0/485 (0)	0.848 (0.437- 1.196)	0.691	11/105 (10)	0.859 (0.790- 0.933)	0.645	2/77 (2)	0.413 (0.324- 0.527)	0.351	0 (0)	0.826 (0.752- 0.907)	0.763
Severe Outcome	41/94 (44)	1.100 (0.704- 1.718)	0.675	14/485 (3)	1.493 (0.282- 2.452)	0.527	0/105 (0)	3.235 (0.671- 15.593)	0.143	26/77 (34)	0.929 (0.392- 2.198)	0.866	9/174 (5)	0.606 (0.203- 1.815)	0.371

*P for comparison between virus-infected (SARI-positive) and non-infected (SARI-negative) individuals. P^$^ for SARI patients with a positive result for that pathogen compared to a reference group of tested SARI patients with a negative result for that pathogen. *RSV* Respiratory syncytial virus, *PIV* Para-influenza virus, *ICU* Intensive care unit, Severe outcome is defined as illness requiring ventilation or ICU or resulting in death

Logistic regression was used to further examine associations with severe outcomes in SARI-positive individuals with complete demographic data and clinical risk factors. By univariate analysis, individuals with positive results for rhinovirus and adults >18 years were more likely to experience a severe outcome than those not infected with rhinovirus (OR 4.975, 95% CI 2.431-17.812, p=0.024) and children <18 years (OR 10.357, 95% CI 5.895-18.197, p=0.000), respectively.

Multivariate analysis confirmed these results where individuals with positive results for rhinovirus and adults >18 years were more likely to experience a severe outcome than those not infected with rhinovirus (OR 4.807, 95% CI 2.981-16.112, p=0.025) and children <18 years (OR 11.716, 95% CI 7.225-18.998, p=0.000), respectively.

Table 3 shows these results.

**Table 3:** Univariate and multivariate logistic regression for predictors of severe outcomes among viral-infected SARI cases

Univariate Analysis			
		OR (95% CI)	P
RSV	Negative	ref	
	Positive	0.00 (−)	0.989
Adenovirus	Negative	ref	
	Positive	0.927 (0.533–1.612)	0.788
Rhinovirus	Negative	ref	
	Positive	4.975 (2.431–17.812)	0.024
Enterovirus	Negative	ref	
	Positive	0.00 (−)	1.000
Influenza	Negative	ref	
	Positive	1.150(0.608–2.176)	0.667
BOCA virus	Negative	ref	
	Positive	0.413 (0.051–3.371)	0.409
HMPV	Negative	ref	
	Positive	0.845 (0.431–1.656)	0.624
PIV	Negative	ref	
	Positive	0.633 (0.361–1.112)	0.112
Multiple viruses	Single virus	ref	
	Multiple viruses	1.515 (0.974–2.357)	0.065
Age	Adults >18 years	ref	
	Children <18 years	10.357 (5.895–18.197)	0.000
Gender	Male	ref	
	Female	0.893 (0.643–1.239)	0.497
Comorbidities	None	ref	
	Any	1.181 (0.840–1.661)	0.338
Multivariate analysis			
		OR (95% CI)	P value
Rhinovirus	Negative	ref	
	Positive	4.807 (2.981–16.112)	0.025
Age	Adults >18 years	ref	
	Children <18 years	11.716 (7.225–18.998)	0.000

*RSV* Respiratory syncytial virus, *hMPV* Human metapneumovirus, *PIV* Para-influenza virus, *OR* Odds ratio

### Comparison between RSV-positive and other viral cases

Being the most commonly detected virus among our cohort, clinical characteristics and outcomes of RSV-positive patients were compared to those with other respiratory positive cases as well as viral-negative patients.

While patients with RSV-positive infections had significant differences with those with no respiratory viruses identified, with regards to clinical signs and symptoms, comorbidities, and outcomes (ICU admission and deaths); they had no differences with those tested positive for other viral pathogens, with regards to the same parameters. (Table 4 shows these details)

**Table 4:** Comparison of SARI patients with RSV to those with a non-RSV or to those with no respiratory virus identified

Characteristic	RSV-positive (n = 485) N (%)	Other Viruses Positive (n = 590) N (%)	P*	No virus Identified (n = 2132) N (%)	P^$^
Gender					
Female	257 (53)	312 (53)	0.203	810 (38)	0.000
Male	228 (47)	278 (47)		1,322 (62)	
Age					
Below 18 y	470 (97)	511 (87)	0.801	1,493 (70)	0.373
Above 18 y	15 (3)	79 (13)		639 (30)	
Symptom onset ≤7 days	456 (94)	480 (81)	0.064	1,211 (57)	0.250
Cough	485 (100)	536 (91)	0.882	2,132 (100)	1.000
SOB	485 (100)	548 (93)	1.000	1,555 (73)	0.077
Fever	485 (100)	590 (100)	1.000	2,132 (100)	1.000
Sore throat	174 (36)	311 (53)	0.000	654 (31)	0.000
Sputum production	254 (52)	312 (53)	1.000	1,169 (55)	0.870
Body aches	60 (12)	105 (18)	0.063	153 (7)	0.004
Tachypnea	442 (91)	558 (95)	0.063	1,732 (81)	0.087
Nasal congestion	322 (66)	325 (55)	1.000	300 (14)	0.060
Wheezing	414 (85)	467 (79)	0.031	360 (17)	0.063
Abnormal BS	239 (49)	319 (54)	0.008	955 (45)	0.022
Nausea or vomiting	54 (11)	62 (10)	0.988	209 (10)	0.003
Convulsions	44 (10)	64 (11)	0.677	63 (3)	0.046
pneumonia	13 (3)	16 (3)	1.000	175 (8)	0.001
Chronic lung disease	227 (47)	233 (39)	0.086	343 (16)	0.001
Cardiac disease	81 (17)	133 (23)	0.063	512 (24)	0.022
Endocrine disease	50 (10)	74 (13)	0.866	147 (7)	0.002
Renal disease	5 (1)	6 (1)	1.000	43 (2)	0.246
Neuromuscular disorder	30 (6)	60 (10)	0.333	176 (8)	0.033
ICU	82 (17)	137 (23)	0.121	606 (28)	0.000
Ventilation	43 (9)	57 (10)	0.473	221 (10)	0.343
ARDS	8 (1)	7 (1)	1.000	3 (0)	0.000
Respiratory Failure	9 (2)	13 (2)	0.988	45 (2)	1.000
Death	12 (2)	12 (2)	1.000	106 (5)	0.000

*P for comparison for SARI patients with a positive result for RSV (RSV-positive SARI patients) and a reference group of tested SARI patients with a negative result for RSV (non-RSV-positive SARI patients).P^$^ for comparison between RSV-positive SARI patients and non-infected individuals (SARI-negative individuals); *RSV* Respiratory syncytial virus, *SOB* Shortness of breath, *ICU* Intensive care unit, *ARDS* Acute respiratory distress syndrome

### Severe outcomes in RSV-positive patients

Logistic regression was used to examine associations with severe outcomes in RSV-positive patients with complete demographic data and clinical risk factors. By univariate analysis, individuals with RSV and associated comorbidities were more likely to experience severe outcomes (OR 4.703, 95% CI 0.803-9.672, p=0.001) than those with RSV and no comorbidities (Table 5).

**Table 5:** Logistic regression for predictors of severe outcomes for RSV-positive cases

Univariate analysis			
		OR (95% CI)	P
Gender	Male	ref	
	Female	1.600 (0.400–6.163)	0.086
Age	Adults >18 years	ref	
	Children <18 years	1.119 (0.276–4.466)	0.151
Comorbidities	None	ref	
	Any	4.703(0.803–9.672)	0.001

*RSV* Repiratory syncytial virus, *OR* Odds ratio

## Discussion

To the best of our knowledge, this is the largest surveillance Egyptian study that addressed the epidemiological patterns of SARI due to viruses and atypical bacteria in both children and adult population and their relation to the clinical characteristics and outcomes of those patients.

The worldwide distribution of viral etiology as a cause of SARI varies between 2% and up to 78% [7, 11, 13, 14]. In this study, we found a viral etiology in 33.5 % of hospitalized patients with SARI, which is comparable to previous studies conducted in either developing or Middle Eastern countries [9–11, 13]. The finding that two-thirds of SARI cases had no pathogen detected suggests that poor or late specimen collection may have contributed to a lower yield of detected viruses. Interestingly, children <18 years represented the majority (91.2%) of our cohort. Notably, this contradicts findings observed by other studies [14, 15]. In their surveillance for SARI in Northern Vietnam, Nguyen *et al* [15] observed that 22.7% of their cohort were children <18 years, while 77.3% were adults >18 years. Again, children <5 years represented 83% of our cases. This is in accordance with those surveillance data from Southern Arizona, 82% [14], lower than those from China (94% in <72 months) [16] and higher than in Kenya,71% [11].

The highest rates of viral infections were reported for RSV (45.2%), PIV (11.6%), and adenovirus (9.8%), with a relatively low rate (7.2%) for influenza viruses. Not unexpected, RSV was the most predominant respiratory virus with a prevalence of 45%; emphasizing its role as the major cause of SARI in infants and young children worldwide [7, 8, 13–17]. Notably, the proportion of SARI cases positive for RSV in children <5 years in our surveillance (90%) was markedly higher than those reported in surveillance data from Kenya, 21% [11], Southern Arizona, 31% [14], and even higher than previous studies in Egypt [18].

We observed that, SARI cases <5 years were significantly more likely than older patients to be infected with each of the pathogens examined, particularly for RSV and influenza. As the majority of enrolled patients were children (83%), this is not unexpected since these pathogens have a strong association with this age group. This is inconsistent with data that nearly 80% of children are exposed to RSV by age two, 100% to hMPV by age five and 90% to hPIV by age five [19]. Furthermore, hPIV is a significant etiology of LRTI in children [20], second only to RSV [21], and adenoviruses are the second most common viral pathogen in children under two years of age [7].

Notably, our results showed a very minor role for atypical bacteria in causing SARI in our locality. Only 3 cases were positive for Mycoplasma (co-infected with RSV), while only one case of Chlamydia was co-infected with RSV and hMPV. Clinical presentations differed significantly between those with non-viral infected individuals and viral-infected SARI ones. The later had significant viral prodromal symptoms, as well as tachypnea, wheezes, and convulsions. Furthermore, SARI patients with influenza had significant tachypnea, wheezes, and abnormal breath sounds, than those with non-influenza viral infections. The presence of these signs at presentation could help the clinician predicting the likely pathogen causing SARI [14].

Fifty-three percent of our patients had medical comorbidities, with the predominance of chronic lung diseases (43%). The impacts of medical comorbidities on patients with SARI were addressed in previous surveillance studies [9, 13, 14]. Despite that 83% of our cohort were children less than 5 years, and patients with comorbidities were significantly older compared to those with no comorbidities, patients with and without viral detection differed significantly in the frequencies of chronic respiratory, as well as endocrine, hepatic and neuromuscular disorders.

Comparing the clinical course, complications, and outcomes between viral-infected cases and non-viral detected controls showed interesting results. Patients with identified viruses had significantly lower rates for ICU admission, hospital stay, length of mechanical ventilation, and overall mortality than those without identified viruses. However, there were no differences with regards to ARDS and mechanical ventilation.

Previous studies showed conflicting results on the impacts of viral infections on clinical outcomes in patients with SARI [9, 13, 14, 19, 22, 23]. Differences in patients’ numbers, enrollment criteria, and methodologies could explain these results. Although PCR has been established as a reliable diagnostic assay with high sensitivity and specificity for respiratory viruses, particularly for RSV [24], the clinical implications of positive laboratory results are still less clear [13].

Patients with positive viral detection had better clinical outcomes than those with no viral detection, in terms of pneumonia, ICU admission, and overall mortality. Furthermore, compared to patients with no virus identified, patients with RSV-positive infection were significantly less likely to have pneumonia, to be admitted to the ICU, mechanically ventilated, and had less mortality.

Interestingly, analyses to assess associations with severe outcomes in the current study revealed that no infections were independently associated with those outcomes, even after controlling for age and associated medical comorbidities. Despite the predominance of RSV infections among SARI-positive cases (45%), there was strong evidence that individuals with RSV and influenza were less likely to experience a severe outcome than those not infected with each of these pathogens. Furthermore, individuals with multiple infections were no more likely than those with infection with a single pathogen to experience severe outcomes.

Multivariate logistic regression analysis confirmed that individuals with positive results for rhinovirus and adults >18 years were more likely to experience a severe outcome than those not infected with rhinovirus and children <18 years, respectively. However, because of the low prevalence of rhinovirus (2%) and adults (8.8%) in this study, further larger studies are needed to confirm these associations.

Being the most commonly detected virus among our cohort, there was an interest to examine the RSV-positive cases. Interestingly, while patients with RSV-positive infections had significant differences with those with no respiratory viruses identified with regards to clinical signs and symptoms, comorbidities, and outcomes; they had no differences with those tested positive for other viral pathogens with regards to the same parameters.

However, individuals with RSV and associated medical comorbidities were more likely to experience severe outcomes than those with RSV and no comorbidities, after controlling for age and other risk factors.

Again, review of the literature had shown conflicting results for clinical implications of RSV infection [9, 23–28]. While the relationship between RSV infection and clinical disease has been established, as infections among asymptomatic individuals are rare [9, 24–27], no relationship between viral load and disease severity was identified by others [23, 28, 29]. For non-influenza viruses, the clinical features are still unclear. Adenovirus infection levels in asymptomatic children and adults varied [27, 30], though this may be attributable to differences in sampling methodology since throat swabs may detect latent AdV DNA in tonsil tissue [27]. Studies suggest that asymptomatic infection with hMPV is rare among children [31], but results from adult populations are less conclusive, with reports of varying levels of infection among asymptomatic individuals [25, 32].

Furthermore, the clinical implications of positive laboratory results are further complicated by the presence of co-infections. Multiple viral respiratory pathogens were identified in 16.7% of our cases. Co-infection with 2 or more viral respiratory pathogens has been encountered in previous reports among pediatric populations in the Middle East [13, 18, 33, 34]. Multiple infections complicate diagnosis, as the relative clinical impact of each pathogen is unclear [13], and certain pathogens, such as adenovirus, are routinely found in the upper airways [35].

This study has many points of strength; it was the first surveillance that addresses the clinical impacts and epidemiological patterns of viral and atypical bacteria causing SARI in both children and adult Egyptian population, with enrolled large numbers of patients and over a relatively long period. Furthermore, analyses of homogenous populations, rather than different ethnic groups [14], give the results reliable and strong support. On the other hand, it has some limitations; more time may be needed for properly evaluating the role of atypical bacteria, and the flu vaccine was not used.

## Conclusions

The current study showed that viral pathogens were encountered in one-third of hospitalized adult and pediatric Egyptian patients with SARI. Atypical bacteria had a minor role in SARI in our locality. Highest rates of viral infections were reported for RSV, PIV, and adenovirus. The presence of chronic respiratory, endocrine, hepatic and neuromuscular disorders negatively affects patients with identified viral infections. Viral infections had no negative impacts on clinical features, clinical course, and severe outcomes of SARI in our locality. Further studies are warranted.

## Abbreviations

ARDS: Acute respiratory distress syndrome
ARIs: Acute respiratory infections
CDC: Center of Disease Control
CUH: Cairo University Hospital
HBOV: Human Bocavirus
hMPV: Human metapneumovirus
Ig: Immunoglobulin
ILI: Influenza-like illness
IQR: Interquartile range
NP: Nasopharyngeal
OP: Oropharyngeal
OR: Odds Ratio
PIV: Parainfluenza virus
RSV: Respiratory syncytial virus
RT-qPCR: Quantitative real-time reverse transcription polymerase chain reaction
SARI: Severe acute respiratory infection
VTM: Viral transport medium
WHO: World Health Organization
